# *In silico-in vitro* modeling to uncover cues involved in establishing microglia identity: TGF-β3 and laminin can drive microglia signature gene expression

**DOI:** 10.3389/fncel.2023.1178504

**Published:** 2023-06-26

**Authors:** Raissa Timmerman, Ella Alwine Zuiderwijk-Sick, Wia Baron, Jeffrey John Bajramovic

**Affiliations:** ^1^Alternatives Unit, Biomedical Primate Research Centre, Rijswijk, Netherlands; ^2^Department of Biomedical Sciences of Cells and Systems, Section Molecular Neurobiology, University Medical Center Groningen, University of Groningen, Groningen, Netherlands; ^3^3Rs Centre Utrecht, Utrecht University, Utrecht, Netherlands

**Keywords:** microglia identity, microglia signature genes, NicheNet, HMGB2, IL-1β, oligodendrocytes, TGF-β3, laminin

## Abstract

Microglia are the resident macrophages of the central nervous system (CNS) and play a key role in CNS development, homeostasis, and disease. Good *in vitro* models are indispensable to study their cellular biology, and although much progress has been made, *in vitro* cultures of primary microglia still only partially recapitulate the transcriptome of *in vivo* microglia. In this study, we explored a combination of *in silico* and *in vitro* methodologies to gain insight into cues that are involved in the induction or maintenance of the *ex vivo* microglia reference transcriptome. First, we used the *in silico* tool NicheNet to investigate which (CNS-derived) cues could underlie the differences between the transcriptomes of *ex vivo* and *in vitro* microglia. Modeling on basis of gene products that were found to be upregulated *in vitro*, predicted that high mobility group box 2 (HMGB2)- and interleukin (IL)-1β-associated signaling pathways were driving their expression. Modeling on basis of gene products that were found to be downregulated *in vitro*, did not lead to predictions on the involvement of specific signaling pathways. This is consistent with the idea that *in vivo* microenvironmental cues that determine microglial identity are for most part of inhibitory nature. In a second approach, primary microglia were exposed to conditioned medium from different CNS cell types. Conditioned medium from spheres composed of microglia, oligodendrocytes, and radial glia, increased the mRNA expression levels of the microglia signature gene *P2RY12.* NicheNet analyses of ligands expressed by oligodendrocytes and radial glia predicted transforming growth factor beta 3 (TGF-β3) and LAMA2 as drivers of microglia signature gene expression. In a third approach, we exposed microglia to TGF-β3 and laminin. *In vitro* exposure to TGF-β3 increased the mRNA expression levels of the microglia signature gene *TREM2*. Microglia cultured on laminin-coated substrates were characterized by reduced mRNA expression levels of extracellular matrix-associated genes *MMP3* and *MMP7*, and by increased mRNA expression levels of the microglia signature genes *GPR34* and *P2RY13*. Together, our results suggest to explore inhibition of HMGB2- and IL-1β-associated pathways in *in vitro* microglia. In addition, exposure to TGF-β3 and cultivation on laminin-coated substrates are suggested as potential improvements to current *in vitro* microglia culture protocols.

## Introduction

Tissue-resident macrophages (TRMs) are innate immune cells that play a role in tissue development, homeostasis, and damage responses through characteristic macrophage functions such as phagocytosis and inflammatory signaling (Davies et al., 2013). In addition to these generic functions, TRM populations vary considerably between tissues in terms of gene expression profiles and fulfill specialized functions, which is partly a consequence of tissue- and niche-specific adaptations (Timmerman et al., 2021). Microglia, the resident macrophages of the central nervous system (CNS), derive from a different progenitor than other TRMs and continuously receive signals from their microenvironment that contribute to their homeostasis (Ginhoux et al., 2010; Bennett et al., 2018). For example, transforming growth factor beta (TGF-β) is constitutively expressed in the CNS and suppresses microglia activation in rodents both *in vitro* and *in vivo* (Suzumura et al., 1993; Butovsky et al., 2014; Zoller et al., 2018). In addition, neighboring neurons express CD47, CD200, and CX3CL1 that interact with CD172, CD200R, and CX3CR1 on microglia, respectively, providing inhibitory signals (Bajramovic, 2011; Szepesi et al., 2018). Loss of constitutive inhibitory signaling leads to a more activated microglia phenotype, characterized by an amoeboid morphology, increased expression of activation markers and loss of microglia signature genes (genes that are highly expressed by microglia and not, or at very low levels, expressed by other macrophages and other CNS cell types) (Hoek et al., 2000; Brionne et al., 2003; Zoller et al., 2018; Baxter et al., 2021). This activated phenotype is also observed when primary microglia are isolated from the CNS environment and are brought in culture (Bohlen et al., 2017; Baxter et al., 2021; Timmerman et al., 2022). Transcriptome studies have demonstrated that the gene expression profile of *in vitro* microglia differs considerably from that of *in vivo* microglia (Butovsky et al., 2014; Bohlen et al., 2017; Timmerman et al., 2022), and analysis of the differentially expressed genes (DEGs) suggests that this is in part attributable to the lack of CNS environmental cues, including the CNS topography, extracellular matrix, and cues from other cell types. This suggestion is strengthened by the observation that loss of microglia signature markers can be partially reversed by engrafting of primary microglia back into a CNS environment or by culturing microglia together with other CNS cell types (Bohlen et al., 2017; Baxter et al., 2021; Timmerman et al., 2022). However, which CNS environmental cues contribute to the *in vivo* microglia gene expression profile that defines their identity is poorly understood.

In this study, we used NicheNet (Browaeys et al., 2020), an *in silico* (computational) method that predicts ligand-target links between interacting cells, to uncover (CNS-derived) ligands that drive the DEGs between *in vivo* and *in vitro* microglia. As a reliable proxy for the transcriptome of *in vivo* microglia, we used an earlier generated reference transcriptome that was directly derived from microglia isolated under cold conditions, *ex vivo* microglia. If possible, we tested the effects of identified candidates and pathways *in vitro.* In a second approach, we exposed microglia to conditioned medium (CM) derived from different CNS cell types and analyzed the effects on microglia signature gene expression. Lastly, we exposed microglia to TGF-β3 and we cultured microglia on laminin-coated substrates, and examined the effects on the expression of microglia signature genes.

## Materials and methods

### Animals

Brain tissue was obtained from adult rhesus macaques (*Macaca mulatta*) of either sex without neurological disease that became available from the outbred breeding colony or from other studies (all studies were ethically reviewed and approved by the Ministry of Agriculture, Nature and Food Quality of the Netherlands). No animals were sacrificed for the exclusive purpose of the initiation of microglia cell cultures. Better use of experimental animals contributes to the priority 3Rs program of the Biomedical Primate Research Centre. Individual identification data of the animals are listed in Table 1.

**TABLE 1:** Individual identification data of rhesus macaques.

Monkey ID number	Age (years)	Sex	Weight (kg)	Origin
R01068	19	M	13	India
R01085	21	F	7	India
R04016	18	F	5	India
R04025	16	F	5	India
R06012	16	F	8	India
R06054	15	F	7	India
R08033	14	F	5	India
R09153	11	M	17	India
R12124	9	F	7	India
R13152	8	M	13	India
R13169	8	M	15	India
R14079	8	F	9	Mix
R14143	6	F	5	India
R15009	6	M	10	India
R15025	6	F	6	India
R17023	5	M	8	India
R17045	3	M	8	India
R18015	3	M	5	India

M, male; F, female.

### Reagents

A total of 5 μM inflachromene (ICM; Cayman Chemical, Ann Arbor, MI, USA), 10 ng/ml human recombinant interleukin (IL)-1β (InvivoGen, San Diego, CA, USA), 250 ng/ml human IL-1 receptor antagonist (IL-1Ra; PeproTech, London, UK), 100 ng/ml lipopolysaccharide (LPS; InvivoGen), 50 ng/ml human transforming growth factor (TGF)-β3 (Miltenyi Biotec, Bergisch Gladbach, Germany).

### Microglia isolation and cell culture

Primary microglia were isolated and cultured as described previously (Timmerman et al., 2022). In short, microglia isolations were initiated from cubes of ∼4.5 g frontal subcortical white matter tissue that were depleted of meninges and blood vessels manually. Tissue was chopped into cubes of less than 2 mm^2^ by using gentleMACS™ C tubes (Miltenyi Biotec) and incubated at 37°C for 20 min in PBS containing 0.25% (w/v) trypsin (Gibco, Life Technologies, Bleiswijk, Netherlands) and 1 mg/ml bovine pancreatic DNAse I (Sigma-Aldrich, Saint Louis, MO, USA) and mixed every 5 min. The supernatant was discarded (no centrifugation), the pellet was washed and passed over a 100 μm nylon cell strainer (Falcon; Becton Dickinson Labware Europe, Vianen, Netherlands) and centrifuged for 7 min at 524 *g.* The pellet was resuspended in 22% (vol/vol) Percoll (Cytiva, Uppsala, Sweden), 37 mM NaCl and 75% (vol/vol) myelin gradient buffer (5.6 mM NaH_2_PO_4_, 20 mM Na_2_HPO_4_, 137 mM NaCl, 5.3 mM KCl, 11 mM glucose, 3 mM BSA Fraction V, pH 7.4). A layer of myelin gradient buffer was added on top, and this gradient was centrifuged at 1,561 *g* for 30 min (minimal brake). The pellet was washed and centrifuged for 7 min at 524 *g.* For laminin coating experiments, tissue culture-treated well plates (Corning Costar Europe, Badhoevedorp, Netherlands) were coated with 10 μg/ml laminin-111 (Sigma-Aldrich) for 2 h at 37°C in a humidified atmosphere containing 5% CO_2_. Plates were washed two times with PBS before seeding the cells. Cells were seeded at a density of 6.5 × 10^4^ cells/cm^2^ in serum-containing microglia medium (SM) comprised of 1:1 v/v DMEM (high glucose)/HAM F10 Nutrient mixture (Gibco) supplemented with 10% v/v heat-inactivated fetal bovine serum (FBS; TICO Europe, Amstelveen, Netherlands), 2 mM glutamax, 50 units/ml penicillin, and 50 μg/ml streptomycin (all from Gibco). After overnight incubation at 37°C in a humidified atmosphere containing 5% CO_2_, unattached cells and debris were removed by washing with PBS twice and replaced by fresh SM medium supplemented with 20 ng/ml macrophage colony-stimulating factor (M-CSF; PeproTech). At day 4, cells were washed twice with PBS and cultivated in serum-free microglia culture medium (SFM) comprised of DMEM/F12 supplemented with 2 mM glutamax, 50 units/ml penicillin, 50 μg/ml streptomycin (all from Gibco), 5 μg/ml N-acetyl cysteine, 5 μg/ml insulin, 100 μg/ml apo-transferrin, 100 ng/ml sodium selenite (all from Sigma-Aldrich), 20 ng/ml M-CSF, 12.5 ng/ml TGF-β1 (Miltenyi Biotec), 1.5 μg/ml ovine wool cholesterol (Avanti Polar Lipids, Alabaster, AL, USA), 1 μg/ml heparan sulfate (Galen Laboratory Supplies, North Haven, CT, USA), 0.1 μg/ml oleic acid and 1 ng/ml gondoic acid (both from Cayman Chemical). All cells were kept in culture for 15 days total without passaging. From day 4 onward, half of the medium was replaced by fresh SFM medium containing new growth factors every 2–3 days. Microglia were exposed to the indicated treatments (ICM, IL-1Ra, and CM) from day 4 onward, which were re-added to the culture medium every 2–3 days during medium changes.

### Oligodendrocyte- and sphere-conditioned medium

Oligodendrocyte-conditioned medium (OCM) was collected from cultured primary rat oligodendrocyte progenitor cells (OPC) or mature oligodendrocytes (mOLG). In short, OPCs were isolated from neonatal non-cortical areas of rat forebrains using a shake-off procedure as described previously (Lentferink et al., 2018; Werkman et al., 2020). The enriched OPC fraction was seeded at a density of 2.1 × 10^4^cells/cm^2^ on 13-mm poly-L-lysine (PLL, 5 μg/ml)-coated glass slides in 24-well plates. Cells were cultured in defined SATO medium comprised of DMEM supplemented with 5 μg/ml bovine insulin, 50 μg/ml human holo-transferrin, 100 μg/ml bovine serum albumin fraction V, 62 ng/ml progesterone, 16 μg/ml putrescine, 5 ng/ml sodium selenite, 400 ng/ml T3, 400 ng/ml T4 (all from Sigma-Aldrich), 4 mM L-glutamine, 100 units/ml penicillin and streptomycin (all from Gibco) and 27.5 μM 2-mercaptoethanol (Sigma-Aldrich). For OPC culture, 10 ng/ml platelet-derived growth factor-AA and 10 ng/ml fibroblast growth factor-2 (both from PeproTech) were added to SATO. For mOLG culture, cells were cultured for 2 days in SATO medium supplemented with PDGF-AA and FGF2, followed by differentiation upon growth factor withdrawal and culturing for 6 days in SATO supplemented with 0.5% FBS. For OCM experiments, OCM was mixed with fresh SFM medium at a volume ratio of 1:2 (OCM:SFM).

Sphere-conditioned medium (SCM) was collected from 3D-spherical co-cultures composed of microglia, oligodendrocytes, and radial glia that were cultivated in SM medium for 4 days, followed by cultivation in SFM medium for 11 days (Timmerman et al., 2022). For experiments where microglia were exposed to SCM, SCM was mixed with fresh SFM medium (vol:vol 1:1).

### NicheNet and ingenuity pathway analysis

**FIGURE 1:**
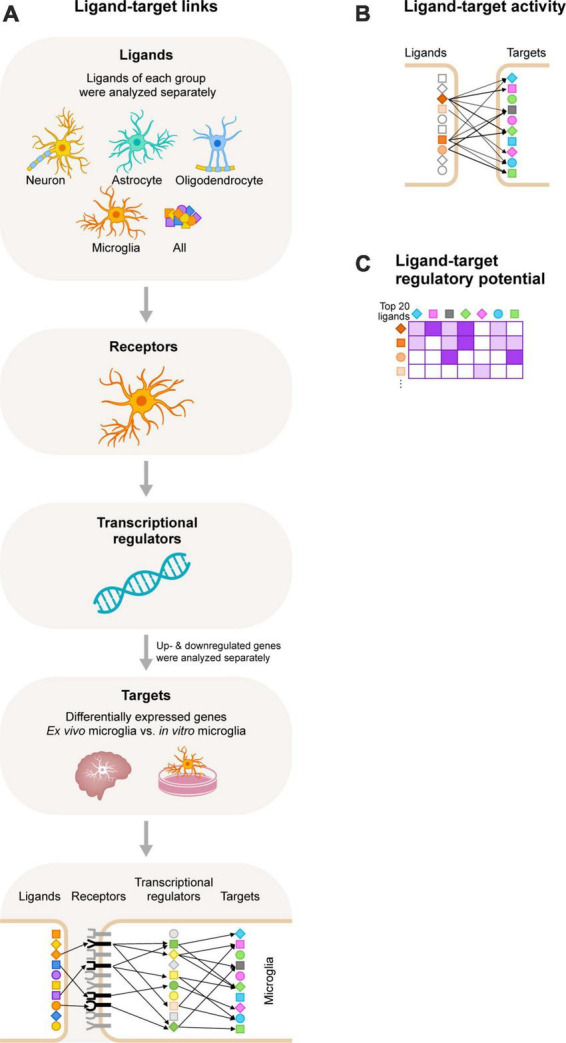
Schematic overview of the NicheNet workflow. **(A)** We selected ligands expressed on neurons, astrocytes, oligodendrocytes, and microglia, and also included an analysis with all ligands of the NicheNet database. Next, we selected receptors expressed by microglia. We used NicheNet’s ligand-receptor data sources to analyze ligand-receptor interactions. Of note, ligands of each group were analyzed separately. Subsequently, information from NicheNet’s signaling data sources were used to analyze which transcriptional regulators are activated by the ligand-receptor interactions. Lastly, NicheNet’s gene regulatory data sources were used to further analyze which target genes (the differentially expressed genes between *ex vivo* and *in vitro* primary microglia from adult rhesus macaques) are regulated by the transcriptional regulators. Of note, upregulated and downregulated genes were analyzed separately. **(B)** After the ligand-target links were determined we performed a ligand-target activity analysis to rank the ligands based on the presence of its target genes. The more target interactions, the higher the ranking of the ligand. **(C)** The 20 ligands with the highest ligand-activity scores based on the presence of their target genes were displayed in the ligand-target heatmaps. In these heatmaps the regulatory potential scores for interaction between the top 20 ligands and target genes is displayed.

The computational tool NicheNet uses gene expression data as input. It combines these with existing ligand-receptor, signaling, and gene regulatory data sources, allowing for predictions on ligand-receptor interactions that drive gene expression changes in cells of interest (Browaeys et al., 2020). We used NicheNet to identify ligands that regulate the expression of DEGs between *in vitro* and *ex vivo* microglia (Figure 1). NicheNet analyses were performed according to the code deposited in GitHub^1^.

For this analysis, we selected ligands expressed on neurons, astrocytes, oligodendrocytes, and microglia, and also included an analysis with all ligands of the NicheNet database. For the expression of neuronal, astrocyte and oligodendrocyte ligands, the adult human transcriptome dataset (GSE73721) of Zhang et al. (2016) was used, where genes with an expression of ≥1.0 FPKM for astrocytes and oligodendrocytes, and ≥0.5 FPKM for neurons in at least one donor were selected. For the expression of microglial ligands and receptors, we used the in-house generated adult rhesus macaque microglia transcriptome dataset (GSE171476) (Timmerman et al., 2022), where genes with an expression of ≥5 CPM in all four *ex vivo* donors were selected. Next, we selected the target genes, which are the DEGs (FC ≥ 4; FDR < 0.01) between *ex vivo* and *in vitro* microglia.

For NicheNet analyses we studied the ligand-target interactions per CNS cell type separately and distinguished between target genes that were significantly upregulated or downregulated *in vitro*. First, we defined a set of potentially active ligands. This are ligands expressed by CNS cell types that can bind to a receptor that is expressed by microglia. These ligand-receptor links were gathered from NicheNet’s ligand-receptor data sources. Second, a ligand-target activity analysis was performed to assess how well ligand-receptor induced activation can predict the expression of the microglial target genes. The 20 ligands with the highest Pearson correlation coefficients (PCCs) (measure used to define ligand-target activity) based on the presence of their target genes were used for further NicheNet analyses. Lastly, ligand-target analyses of the top 20 ligands were performed and displayed in the ligand-target heatmap. In these heatmaps the regulatory potential scores for interaction between the top 20 ligands and their target genes are displayed. Of note, in the ligand-target heatmaps, we show regulatory potential scores for interactions between the 20 top-ranked ligands and the 250 most strongly predicted targets of at least one of the 20 top-ranked ligands. In addition, regulatory potential scores were set as 0 if the score was below a predefined threshold, which was here the 0.25 quantile of scores of interactions between the ligands and each of their respective top targets. Some regulatory potential scores were below the predefined threshold for some ligands and its targets. As a consequence, these ligands and targets were removed from the heatmaps. NicheNet does not process information on whether interactions are positively or negatively regulated. To accommodate for that information, we used Ingenuity Pathway Analysis (IPA) (Spring release March 2020; QIAGEN^2^) to reveal the signaling networks and signaling regulations of the ligand-target interactions.

### RNA extraction and quantitative RT-PCR

Total cellular RNA was isolated using the RNeasy minikit (Qiagen) according to manufacturer’s protocol. Subsequently, mRNA was reverse transcribed into cDNA using the RevertAid First Strand cDNA synthesis kit according to the manufacturer’s protocol (Fermentas; Thermo Fisher Scientific, Waltham, MA, USA). RT-PCRs were performed on the CFX96™ Real-Time PCR detection system (Bio-Rad Laboratories, Hercules, CA, USA) using primer (Invitrogen; Life technologies) and probe (human Exiqon probe library, Roche, Woerden, Netherlands) combinations listed in Table 2, and iTaq Universal Probes Supermix (Bio-Rad). Relative mRNA expression was standardized to housekeeping gene ACTB using the Pfafll method (Pfaffl, 2001).

**TABLE 2:** Overview of primer/probe combinations used for RT-PCR.

Gene name	Forward primer (5′-3′)	Reverse primer (5′-3′)	Probe
ACTB	GCCCAGCACGATGAAGAT	CGCCGATCCACACAGAGTA	AGGAGGAG
CCL2	CAGCACTTCTGTGCCTGCT	GGGGCATTGATTGCATCT	GGCTGAAG
CCNF	GGGAAGATTCGAGTCCCCAC	GTGCAGCAGGGAGAGCTC	CAGAGGAA
CX3CR1	TGATTTTCCTGGAGACGCTTA	TCAGATCCCTCCTCATGTCA	TTCCCAGT
E2F7	GCTCGCCATGGTTCTTTCAA	AGTAGCCACCTGATCCTTGT	CCCAGCAG
FOS	GGGATGGCATCAAGGTACCC	CCCTTCTTCCCCTCCGAAAC	TCTGGAGC
GPR34	TGACGACAACTTCAGTCAGCA	GGTTGGTCGCTATGACTGGT	CTCCTCCC
IL6	ACAAAAGTCCTGATCCAGTTCC	GTCATGTCCTGCAGCCACT	CAGCAGGC
IL12p40	CCACATTCCTACTTCTCCCTGA	ACCGTGGCTGAGGTCTTGT	TCCAGGTC
KIF2C	GACACATACTATGGGCGGAGA	CCGGTAGCAGGGTTGATTCT	CTTCCTCC
LCN2	CCCAGGACTCCAGCTCAG	CATACCACTTCCCCTGGAAC	TCTGCTGC
MKI67	ACACTCCACCTGTCCTGAAGA	GTGCCTTCACTTCCACACTG	TGAGGCTGT
MMP3	CCTGACGTTGGTCACTTCAC	AATCTCGTGTATAATTCACAATCCTG	TTCCTGGC
MMP7	GCTCATGCCTTTGCACCT	GCGTTGCAGCATACAGGA	CTCCTCCA
MMP9	ACAAGCTCTACGGCTTCTGC	GAAGGTGAAGGGGAAAACG	CAGCTCCC
MMP14	CCAAGACCCTCCCGTTGT	GGCAGGTAGCCATACTGCTG	GGGAGCAG
P2RY12	TCCATTCAAAATTCTTAGTGATGC	CGGAGGTAACTTGACACACAAA	TTCCCAGT
P2RY13	ACTGAGTATCCTCCCAAAGGTG	CGGTCAAGAAAACCACTGTGT	CCCAGCCC
PHF19	TGGAAGGACATACAGCATGC	ACACTTCCCGCAGATGAGGA	CTTCCCCA
SHCBP1	GCAATTGAGCATGTCAGATTTTTC	CGAGGTTCAACACATCTGACA	CATCCTCC
TACC3	CCAGAAAGCCCTGAGACCA	TCCGCTGAGGCTGAATGCAG	GGAGCCAG
TNF-α	AAGCCTGTAGCCCATGTTGT	GCTGGTTATCTGTCAGCTCCA	CCAGGAGG
TREM2	CCGGCTGCTCATCTTACTCT	AGGACACCTGTAGGGACTGG	TCTGGAGC
TYMS	AAAACCAACCCTGACGACAGA	CACCACATAGAACTGGCAGAG	CTGCCTCC

### Statistics

GraphPad Prism 9.2.0 (GraphPad Software, San Diego, CA, USA) was used for statistical analysis. Statistical details of experiments can be found in the figure legends.

## Results

### *In silico* modeling of DEGs that were upregulated *in vitro* predicts that HMGB2- and IL-1β-associated signaling pathways are overactivated in primary microglia

We used NicheNet (Browaeys et al., 2020), an *in silico* tool to study intercellular communication, to uncover CNS-derived cues that contribute to the gene expression profile that is characteristic for *in vivo* microglia. NicheNet uses gene expression data as input and combines these with existing ligand-receptor, signaling and gene regulatory data sources, allowing for predictions on ligand-receptor interactions that drive gene expression changes in cells of interest (Figure 1).

We started our analyses by filtering for (i) ligands expressed by CNS cells (neurons, astrocytes, oligodendrocytes, and microglia), (ii) receptors expressed by microglia, and (iii) target genes, which are the DEGs between *ex vivo* and *in vitro* microglia (Timmerman et al., 2022). We then performed NicheNet analyses for each CNS cell type separately, and for the up- and downregulated target genes separately. We also included an unbiased analysis where all ligands of the NicheNet database were used to include possible CNS intercellular signaling that has not been described yet, or that are mediated by other CNS cells such as OPC. A schematic overview of the NicheNet workflow is shown in Figure 1A. Next, we performed a ligand-target activity analysis to rank ligands based on the presence of its target genes (Figure 1B). We selected the top 20 ligands with the highest PCCs (measure of the ligand-target activity), and analyzed the regulatory potential scores for the top 20 ligands to interact with the target genes (Figure 1C). These scores were calculated from interactions inferred from several complementary ligand-receptor, signaling and gene regulatory data sources. Of note, in the ligand-target heatmaps, we show regulatory potential scores for interactions between the 20 top-ranked ligands and the 250 most strongly predicted targets of at least one of the 20 top-ranked ligands. In addition, a predefined threshold was set for the regulatory potential scores. As a consequence, some ligands and/or target genes were removed from the ligand-target heatmaps (see section “Materials and methods”). The remaining ligands and their PCC scores and regulatory potential scores for the up- or downregulated targets are displayed in Supplementary Figures 1–3, respectively. We observed that the PCC scores of the ligands and the downregulated genes *in vitro* were very low (<0.05; Supplementary Figure 1). This implies that the ranking of the ligands would not be much better than random prediction (Browaeys et al., 2020). For this reason, we decided not to continue with the analysis of the downregulated target genes and to focus on the ligands that are predicted to drive the expression of the upregulated target genes, as the PCC scores of these ligands were much higher.

**FIGURE 2:**
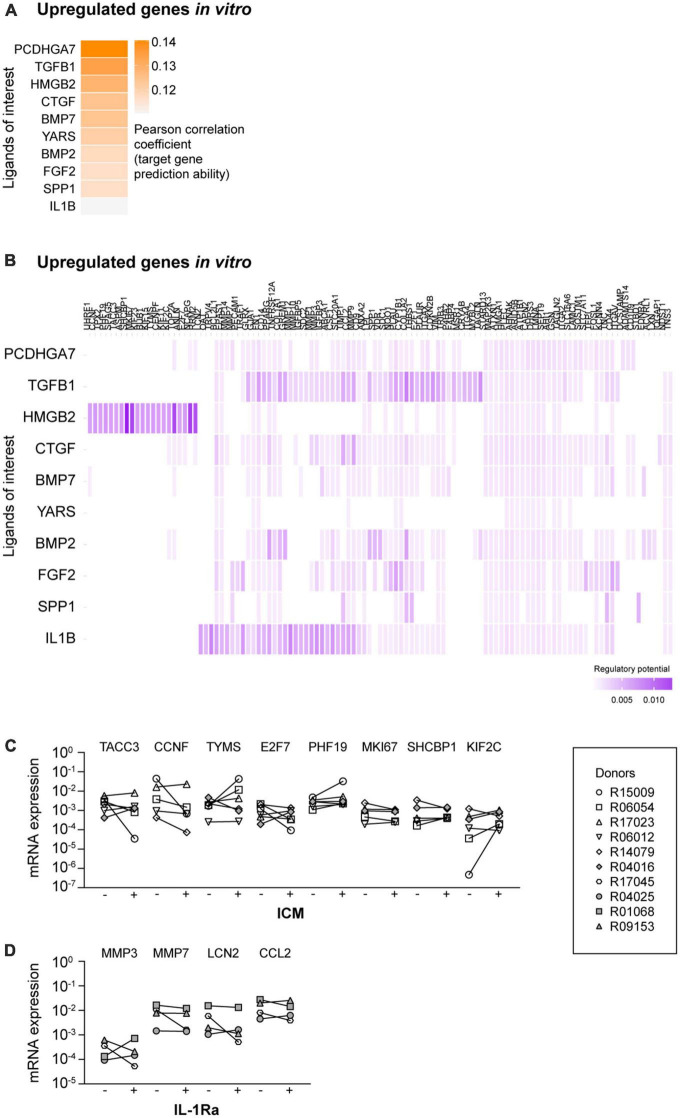
NicheNet analyses of the ligands of interest (LOI) and the upregulated target genes. **(A)** Ligand-target activity analysis of the LOI and the upregulated target genes. **(B)** Ligand-target matrix denoting the regulatory potential between the LOI and the upregulated target genes. mRNA expression levels of upregulated target genes in the presence of **(C)** inflachromene (ICM) and **(D)** interleukin 1 receptor antagonist (IL-1Ra). –, mRNA expression in the absence of the inhibitor; +, mRNA expression in the presence of the inhibitor. Symbols represent different donors, *n* = 4–6 dependent on the inhibitor, paired *t*-test on log-transformed data.

When we compared all ligand-target heatmaps of the upregulated *in vitro* target genes, we observed that some predicted ligands were present in multiple heatmaps. Furthermore, some target genes were predicted to be regulated by multiple upstream ligands (Supplementary Figure 2). For this reason, we selected the 10 ligands from the ligand-target heatmaps that together regulate most target genes. Of note, as multiple ligands regulate the same target genes, we also took the regulatory potential scores into account. We named these selected ligands: ligands of interest (LOI; Supplementary Table 1) and performed new NicheNet analyses with these ligands (Figure 2A).

High mobility group box 2 (HMGB2) was predicted as an upstream regulator for a specific selection of upregulated *in vitro* target genes (Figure 2B). The majority of the other LOI were predicted to regulate almost all remaining target genes in the heatmap. TGF-β1 showed high potential regulatory scores. However, this factor is already present in our culture medium. As the regulatory potential scores of IL-1β and its target genes were higher compared to the other ligands, we selected HMGB2 and IL-1β as potential ligands that regulate the expression of the upregulated genes *in vitro*. As NicheNet does not provide information on whether predicted ligands regulate their associated targets positively or negatively, we used QIAGEN IPA to examine this. IPA did not confirm all NicheNet’s predicted ligand-target interactions, but did predict that inhibition of HMGB2- (Supplementary Figure 4) and IL-1β-mediated signaling (Supplementary Figure 5) would reduce the mRNA expression levels of most target genes.

### Other factors than extracellular HMGB and IL-1 are responsible for the upregulated target gene expression profile in primary microglia

The predicted activation of HMGB2- and IL-1β-associated pathways is in line with the idea that *in vitro* microglia are overactivated in culture (Bohlen et al., 2017; Cadiz et al., 2022). Whether direct exposure of microglia to HMGB2 and/or IL-1β is indeed responsible for the overactivation of these damage-associated pathways is unknown. To test this, we examined the effects of inhibiting HMGB2- and IL-1β-mediated signaling in cultured primary microglia.

To inhibit HMGB2-mediated signaling, we added ICM, a recently described inhibitor of both HMGB1 and HMGB2, to the microglia culture medium (Lee et al., 2014). Originally, HMGBs have been described as DNA-binding proteins (reviewed in Stros, 2010). However, more recent studies have demonstrated that HMGBs can also be released into the extracellular space and can interact with various receptors to activate inflammatory pathways (Wang et al., 1999; Scaffidi et al., 2002; Pusterla et al., 2009; Lee et al., 2014). We confirmed the activity of ICM by demonstrating that ICM exposure reduced the mRNA expression levels of LPS-induced pro-inflammatory cytokines IL-6, IL-12p40, and TNF-α (Supplementary Figure 6A), as reported by Lee et al. (2014). Subsequently, we analyzed the mRNA expression levels of *TACC3, CCNF, TYMS, E2F7, PHF19, MKI67, SHCBP1*, and *KIF2C*, as selected target genes of which the expression was predicted to be regulated by HMGB2-mediated signaling (Figure 2C), and observed that exposure to ICM did not affect the mRNA expression levels of these genes.

To inhibit IL-1β-mediated signaling, cultured primary microglia were exposed to IL-1Ra. Importantly, IL-1Ra inhibits the activity of both extracellular IL-1α and IL-1β by competitively blocking their binding to type I and type II receptors (Rosenzweig et al., 2014). The activity of IL-1Ra was confirmed by demonstrating that IL-1β-induced IL-6 mRNA expression levels were reduced in the presence of IL-1Ra (Supplementary Figure 6B; Lee et al., 2004). We analyzed the mRNA expression levels of *MMP3, MMP7, LCN2*, and *CCL2*, as selected target genes of which the expression was predicted to be regulated by IL-1β-mediated signaling. However, we did not observe a reduction in mRNA expression levels of these target genes in the presence of IL-1Ra (Figure 2D).

Together, these data demonstrate that non-specific inhibition of HMGB2- and IL-1β-mediated signaling does not reduce the mRNA expression levels of the analyzed target genes. This suggests that other ligands are responsible for the overactivation of HMGB2- and IL-1β-associated signaling in *in vitro* microglia.

### Exposure to conditioned medium of mixed glia cell spheres increased the mRNA expression levels of P2RY12 in primary microglia

**FIGURE 3:**
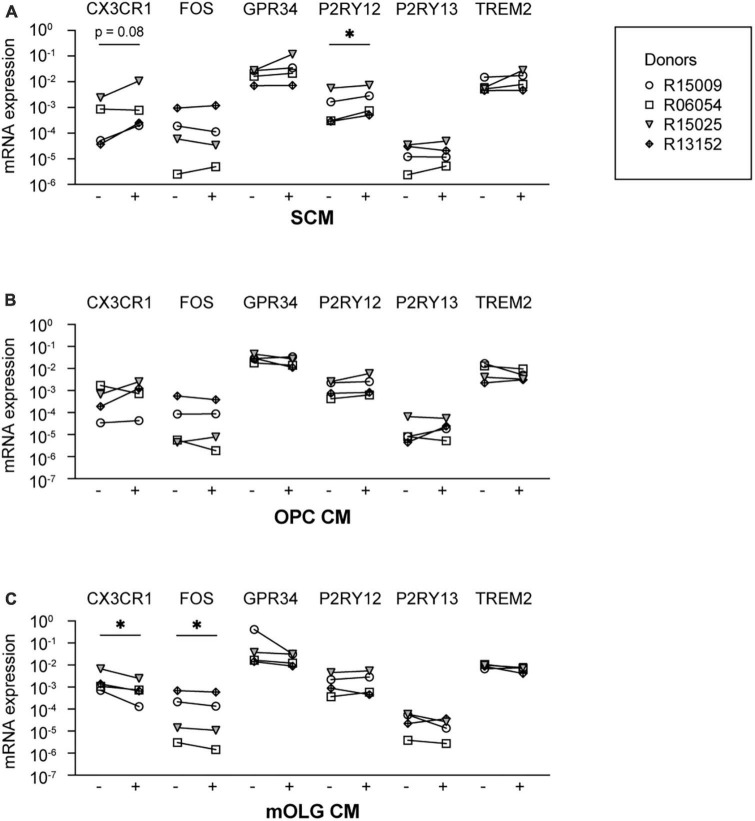
Exposure to conditioned medium of mixed glia cell spheres increased the mRNA expression levels of P2RY12. Microglia culture medium was supplemented with conditioned medium from either **(A)** spheres composed of microglia, oligodendrocytes, and radial glia (SCM), **(B)** rat oligodendrocyte precursor cells (OPC CM), or **(C)** rat mature oligodendrocytes (mOLG CM). Subsequently, mRNA expression levels of microglia signature genes were measured using RT-PCR. –, mRNA expression in the absence of conditioned medium; +, mRNA expression in the presence of conditioned medium. Symbols represent different donors, *n* = 4, paired *t*-test on log-transformed data, *p < 0.05.

Earlier data from our group demonstrated that microglia cultured in spheres, together with oligodendrocytes and radial glia, are characterized by the increased expression of microglia signature genes (Timmerman et al., 2022). Therefore, in a second approach to gain insight into cues that could potentially optimize microglia *in vitro* culture conditions, we exposed microglia to SCM, to determine if these effects were attributable to factors secreted from cells in the spheres. We analyzed the mRNA expression levels of the microglia signature genes *CX3CR1, FOS, GPR34, P2RY12, P2RY13*, and *TREM2*, as their expression was increased in spheres as compared to monocultured microglia (Supplementary Figure 7). Exposure to SCM indeed increased the mRNA expression levels of *P2RY12*, and showed a trend toward increased mRNA expression levels of *CX3CR1* (Figure 3A).

Since, after microglia, oligodendrocytes are the most abundant cell type in the spheres (Timmerman et al., 2022), we examined whether secreted factors by cultured oligodendrocytes could induce the expression of microglia signature genes. We therefore supplemented the microglia culture medium with CM derived from cultured primary rat oligodendrocyte progenitor cells (OPC CM) or from cultured primary rat mature oligodendrocytes (mOLG CM). We found that neither exposure to OPC CM (Figure 3B) nor to mOLG CM (Figure 3C) led to an increase of microglia signature gene expression levels. Exposure to mOLG CM even led to significantly reduced mRNA expression levels of *CX3CR1* and *FOS*.

### Exposure to TGF-β3 increased the mRNA expression levels of the microglia signature gene TREM2 in primary microglia

**FIGURE 4:**
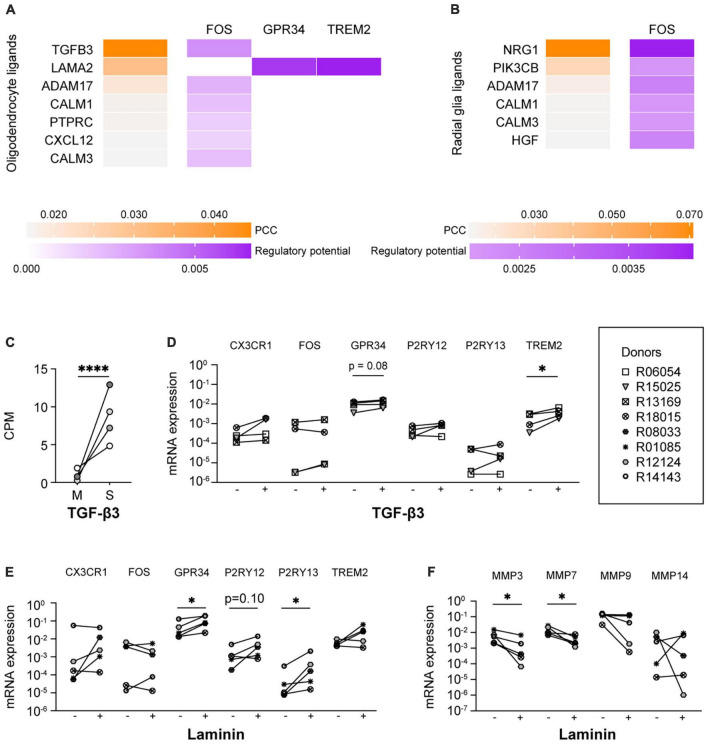
TGF-β3 and laminin exposure increased the mRNA expression of microglia signature genes. Ligand-activity and ligand-target matrix of **(A)** oligodendrocyte ligands and **(B)** radial glia ligands. PCC, Pearson correlation coefficient. **(C)** Gene expression (CPM) of transforming growth factor beta 3 (TGF-β3) in monocultured microglia (M) and spheres (S), *n* = 4, EdgeR false discovery rate (FDR) was used to display statistical differences, ****FDR < 0.001. **(D)** mRNA expression levels of microglia signature genes of microglia cultured in the absence (–) or presence (+) of TGF-β3. Symbols represent different donors, *n* = 4, paired *t*-test on log-transformed data, *p < 0.05. mRNA expression levels of **(E)** microglia signature genes and **(F)** matrix metalloproteinases of microglia cultured in the absence (–) or presence (+) of laminin. Symbols represent different donors, *n* = 5, paired *t*-test on log-transformed data, *p < 0.05.

In a further attempt to identify the ligands present in spheres that were responsible for the induction of microglia signature genes, we performed a NicheNet analysis with expressed ligands in human oligodendrocytes (Zhang et al., 2016) and human radial glia (Pollen et al., 2015), and the six microglia signature genes as target genes. NicheNet did not predict that ligands expressed by oligodendrocytes or radial glia were drivers of *CX3CR1* or *P2RY12* expression levels (Figures 4A, B), which would be in line with the idea that their expression is regulated by microglial contact with other CNS cell types (Szepesi et al., 2018; Zhang et al., 2019; Liu and Aguzzi, 2020; Matejuk and Ransohoff, 2020; Baxter et al., 2021). Nevertheless, multiple oligodendrocyte and radial glia ligands, including TGF-β3, were predicted to drive the expression of *FOS*. IPA analysis confirmed this and further predicted that TGF-β3 positively regulates the expression of *FOS* (Supplementary Figure 8). TGF-β3 is an interesting ligand in this context, since the expression levels of TGF-β3 were significantly higher in the spheres as compared to monocultured microglia (Figure 4C; Timmerman et al., 2022). This in contrast to NRG1, a ligand expressed by radial glia that is also predicted to regulate the expression of FOS, which was not significantly upregulated in spheres when compared to monocultured microglia. Supplementation of the culture medium with TGF-β3 led to increased mRNA expression levels of *TREM2* and showed a trend toward increased expression of *GPR34* (Figure 4D). Of note, TGF-β1, another member of the TGF-β family, is already present in our standard culture medium.

### Laminin coating reduced the mRNA expression levels of matrix metalloproteinases and increased the mRNA expression levels of microglia signature genes in primary microglia

Interestingly, the non-soluble oligodendrocyte ligand LAMA2 was predicted by NicheNet to drive the expression of *GPR34* and *TREM2* (Figure 4A). The gene *LAMA2* encodes for a laminin subunit protein (Aumailley, 2013), which is part of the brain extracellular matrix (bECM) (Jucker et al., 1996). We therefore analyzed the expression levels of microglia signature genes of microglia cultured on laminin-coated substrates. As predicted, the mRNA expression levels of *GPR34* were indeed significantly increased, as were the mRNA expression levels of *P2RY13* (Figure 4E). In addition, we observed a trend toward increased expression of *P2RY12.* Although laminin has been described to be able to affect *in vitro* microglia morphology (Chamak and Mallat, 1991; Tam et al., 2016), we observed no additional effect of laminin on the percentage of ramified microglia or on the numbers of microglia primary branches (Sharaf et al., 2022). It is noteworthy in this context that our primary microglia were already highly ramified.

Interestingly, the expression of several matrisome genes is highly increased in *in vitro* microglia compared to *ex vivo* microglia (Supplementary Figure 9; Timmerman et al., 2022). This is consistent with the reported increased expression of matrisome genes, including cathepsins and matrix metalloproteinases (MMPs), during microglia activation (del Zoppo et al., 2007; Lively and Schlichter, 2013; Nakanishi, 2020). As MMPs were among the ECM-associated genes with the most robust increased expression in *in vitro* microglia, we analyzed the effect of laminin on the mRNA expression levels of MMP genes. Indeed, we found that the mRNA expression levels of *MMP3* as well as *MMP7* were reduced when microglia were cultured on laminin-coated substrates (Figure 4F).

## Discussion

In this study, we combined *in silico-in vitro* modeling to uncover cues that drive the expression of DEGs between *ex vivo* and *in vitro* primary microglia, with a specific focus on microglia signature genes. As total ligand-receptor interaction (the interactome) data at the protein level of *in vitro* microglia are currently not available, we chose to use an RNA transcriptome approach, followed by *in silico* analysis (NicheNet). Our aim was not only to gain insight into the determinants of microglial identity, but also to implement this knowledge to optimize microglia cell culture protocols.

Results from our first approach using the *in silico* tool NicheNet in combination with IPA analysis predicted that HMGB2- and IL-1β-associated signaling pathways are overactivated in primary microglia. HMGB2 and IL-1β are both associated with microglia pro-inflammatory processes (Pinteaux et al., 2002; Lee et al., 2014; Liu and Quan, 2018; Wu et al., 2021) and overactivation of these damage-associated pathways is in line with studies demonstrating that pro-inflammatory gene products are upregulated *in vitro* (Bohlen et al., 2017; Cadiz et al., 2022).

In this study, ICM and IL-1 receptor antagonist (IL-1Ra) were used to test if exposure to HMGB2 and IL-1β indeed underlie the overactivation of the HMGB2- and IL-1β-associated pathways, respectively. It is important to note that ICM binds to both HMGB1 and HMGB2 (Lee et al., 2014), and that IL-1Ra inhibits the activity of both IL-1α and IL-1β by competitively blocking their binding to type I and type II receptors (Rosenzweig et al., 2014). Neither inhibition of HMGB- nor of IL-1R-mediated signaling did reduce the mRNA expression levels of the predicted target genes. This may be attributable to the lack of specificity of the inhibitors used. An alternative explanation for the overactivation of HMGB2- and IL-1β-associated pathways in primary microglia is the absence of CNS-associated inhibitory signaling *in vitro* as suggested before (reviewed in Biber et al., 2007; Kierdorf and Prinz, 2013). This is also in line with the fact that the NicheNet results for the DEGs that were downregulated *in vitro* were challenging to interpret due to the low PCC scores, suggesting that activating signals play overall a less prominent role in the maintenance of a homeostatic microglial profile. Regardless of the exact cause of overactivation of HMGB2- and IL-1β-associated pathways, our results suggest that reversing the overactivated state in primary microglia would improve *in vitro* modeling. Whether this can be best achieved by direct – intracellular – inhibitory targeting of overactivated pathways or by exposure to extracellular neuro-immune-regulators (NIREGs), such as CD200, CD47, and CX3CL1 (Griffiths et al., 2007; Bedoui et al., 2018) remains to be determined. In addition, it is worth mentioning that NicheNet is still relatively immature, and its usefulness for studying complex systems like the CNS remains to be assessed in other studies.

In a second approach to optimize microglia *in vitro* culture conditions, we experimented with exposure to CM derived from different CNS cell types. We had demonstrated in a previous study that microglia cultured in spheres, together with oligodendrocytes and radial glia, were characterized by increased expression levels of microglia signature genes (Timmerman et al., 2022). Exposure to conditioned medium of these spheres (SCM) increased the mRNA expression levels of microglia signature gene *P2RY12* and showed a trend toward increased expression of *CX3CR1*. Interestingly, the expression of *P2RY12* and *CX3CR1* is thought to be regulated by neuron-microglia, astrocyte-microglia and OPC-microglia crosstalk (Szepesi et al., 2018; Zhang et al., 2019; Liu and Aguzzi, 2020; Matejuk and Ransohoff, 2020; Baxter et al., 2021). Our results suggest a novel role for oligodendrocyte- and/or radial glia-secreted factors in the regulation of these genes. Of note, the spheres (Ø ± 250 μm) could only be generated when cultured in relatively high volumes (2.5 ml), and the secreted factors were therefore considerably diluted. The effects of the secreted factors on microglia present in the spheres might therefore be much more robust as those observed in the SCM experiments. Further research is needed to examine if exposure to, e.g., concentrated SCM generates more positive effects. While not present in the spheres, it would be interesting to investigate the role of primate-derived neurons and astrocytes (CM) on microglia homeostasis as well, as these cells promote microglia homeostasis in rodents (Baxter et al., 2021).

Although exposure to CM is a useful approach to analyze the effects of cell-secreted cues on microglia signature gene expression, CM is less suitable as a supplement for cell culture. CM is notoriously associated with batch-to-batch variation, and as long as the components in CM are not defined, standardization of culture conditions will remain challenging. We performed new NicheNet analyses to gather more detailed information about ligands expressed by human oligodendrocytes and radial glia with the potential to regulate the expression of microglia signature genes. NicheNet predicted multiple oligodendrocyte and radial glia ligands as drivers of the microglia signature gene *FOS*, including TGF-β3, ADAM17, NRG1, and PIK3CB. Although the TGF-β1- and TGF-β2-mediated signaling pathway has been reported earlier as an important driver of microglia homeostasis and identity (Brionne et al., 2003; Butovsky et al., 2014; Zoller et al., 2018; Zhang et al., 2019; Liu and Aguzzi, 2020; Baxter et al., 2021), TGF-β3 is a new and interesting candidate. Exposure to TGF-β3 did increase the mRNA expression levels of *TREM2* and did show a trend toward increased expression of *GPR34.* Of note, TGF-β1 was already present in our standard culture medium. There are three known isoforms of TGF-β (TGF-β1, TGF-β2, and TGF-β3) and they are all expressed in the human brain (De Groot et al., 1999). TGF-β1 and TGF-β2 share 71% protein sequence similarity (ten Dijke et al., 1988), whereas TGF-β3 shares 80% of amino acid sequence with TGF-β1 and TGF-β2. Although all isoforms function through the same receptor signaling pathways (Cheifetz et al., 1987; Mittl et al., 1996), biological activity differences between TGF-β isoforms are described (Huang et al., 2014), and it is unclear if these differences affect microglia homeostasis. As far as we are aware of, microglia culture medium is supplemented with either isoform TGF-β1 or TGF-β2 (Butovsky et al., 2014; Bohlen et al., 2017; Gosselin et al., 2017; Baxter et al., 2021; Timmerman et al., 2022). It warrants further investigation if exposure to combinations of different TGF-β isoforms affects TGF-β-mediated signaling differently compared to exposure to each isoform alone, such as described for other agonist isoforms after binding to their associated receptors (Alok et al., 2017; Peach et al., 2018). In addition, it should be tested whether exposure to higher concentrations of TGF-β1 or TGF-β3 could simulate the effects of co-exposure to TGF-β1 and TGF-β3.

Recently, a role for OPCs in maintaining the microglia homeostatic state has been described in rodents (Zhang et al., 2019; Liu and Aguzzi, 2020). However, we observed that exposure to OPC- or mOLG-CM did not induce the mRNA expression levels of microglia signature genes. Of note, these oligodendrocytes were derived from rat, and it is not known if rat- and rhesus-derived oligodendrocytes secrete similar factors. In addition, the indirect effects that other cells in the spheres might have had on the oligodendrocyte secretome could not be tested either.

NicheNet further predicted the oligodendrocyte ligand LAMA2, a laminin subunit, as a driver of *GRP34* and *TREM2* expression. Although laminin is mainly expressed by endothelial cells, astrocytes, and mural cells (reviewed in Kang and Yao, 2022), recent RNA-seq studies have demonstrated that oligodendrocytes express laminin gene products, including LAMA2^3^,^4^ (Zhang et al., 2016; He et al., 2018; Vanlandewijck et al., 2018). We therefore cultured microglia on laminin-coated substrates and demonstrate that they were characterized by increased mRNA expression levels of microglia signature genes and by reduced mRNA expression levels of MMPs. It should be noted though that laminin-111 (previous named laminin-1) with the chain composition α-1/β-1/γ-1 was used in our experiments [for the most recent laminin nomenclature (see, Aumailley et al., 2005)] whereas LAMA2 is a subunit of laminin-211 and laminin-221. Although laminin-111, laminin-211, and laminin-221 can all bind to integrin α6β1, which is expressed in rhesus macaque primary microglia (GSE171476) (Timmerman et al., 2022), laminin-211 and laminin-221 can also bind to integrin α7β1 and dystroglycan. Although ITGA7, the mRNA encoding integrin subunit alpha 7, is not expressed in rhesus primary microglia, DAG1, the gene product of dystroglycan, is expressed. It might therefore be worthwhile to further explore whether LAMA2-mediated signaling has additional effects on the transcriptome of primary microglia.

Taken together, our results suggest that HMGB2- or IL-1β-associated pathways are targets to specifically inhibit upregulated genes *in vitro* to better mimic *in vivo* microglia. In addition, exposure to TGF-β3 and cultivation on laminin-coated substrates are suggested as improvements over current culture practices. Importantly, as the transcriptomes of primary microglia and stem cell-derived microglia are highly similar (Brownjohn et al., 2018; Gumbs et al., 2022), our data might also be applicable to optimize cell culture conditions of stem cell-derived microglia.

## Data availability statement

The original contributions presented in this study are included in the article/Supplementary material, further inquiries can be directed to the corresponding author.

## Ethics statement

The animal study was reviewed and approved by the Ministry of Agriculture, Nature and Food Quality of the Netherlands. Brain tissue was obtained from adult rhesus macaques (*Macaca mulatta*) of either sex without neurological disease that became available from the outbred breeding colony or from other studies. No animals were sacrificed for the exclusive purpose of the initiation of microglia cell cultures. Better use of experimental animals contributes to the priority 3Rs program of the Biomedical Primate Research Centre.

## Author contributions

RT and JB: conceptualization. RT, WB, and EZ-S: experimental work. RT, EZ-S, and JB: data analysis. RT, WB, and JB: manuscript. All authors contributed to the article and approved the submitted version.

## Footnotes

1
https://github.com/saeyslab/nichenetr

2
https://www.qiagen.com/us/products/discovery-and-translational-research/next-generation-sequencing/informatics-and-data/interpretation-content-databases/ingenuity-pathway-analysis

3
https://www.brainrnaseq.org

4
http://betsholtzlab.org/VascularSingleCells/database.html
