# Supplementary material for: In silico-in vitro modeling to uncover cues involved in establishing microglia identity: TGF-β3 and laminin can drive microglia signature gene expression
Source: Front Cell Neurosci. 2023 Jun 26;17:1178504. doi: 10.3389/fncel.2023.1178504 (PMC10330817; doi:10.3389/fncel.2023.1178504)
Supplement: Supplementary file 1 [file Data_Sheet_1.docx]

Supplementary Material

In silico-in vitro modeling to uncover cues involved in establishing microglia identity: TGF-β3 and laminin can drive microglia signature gene expression

Raissa Timmerman^1^, Ella Zuiderwijk-Sick^1^, Wia Baron^2^, Jeffrey Bajramovic^1,3,*^

^1^Alternatives Unit, Biomedical Primate Research Centre, Rijswijk, The Netherlands

^2^Department of Biomedical Sciences of Cells & Systems, Section Molecular Neurobiology, University of Groningen, University Medical Center Groningen, Groningen, the Netherlands

^3^3Rs Centre Utrecht, Utrecht University, Utrecht, The Netherlands

^*^Corresponding author: J.J.Bajramovic@uu.nl

**Figure S1. NicheNet upstream ligand-target activity analysis for differentially expressed genes between *ex vivo* and *in vitro* microglia.** Top potential upstream ligands expressed by either **(A)** neurons, **(B)** astrocytes, **(C)** oligodendrocytes or **(D)** microglia based on their potential to regulate significantly (FC ≥ 4; FDR < 0.01) upregulated (left) or downregulated (right) genes between *ex vivo* and *in vitro* primary microglia. **(E)** We also included an unbiased approach where all ligands of the database were used. As the ligand-target activity ranking metric, the Pearson correlation coefficient is used. This metric indicates the ability of each ligand to predict the expression of the differentially expressed genes, and better predictive ligands are thus ranked higher.

**Figure S2. Ligand-target network matrix of the top-ranked ligands that potentially regulate genes upregulated in *in vitro* microglia compared to *ex vivo* microglia.** NicheNet’s ligand-target matrix denoting the regulatory potential between ligands expressed by **(A)** neurons, **(B)** astrocytes, **(C)** oligodendrocytes or **(D)** microglia, and significantly upregulated *in vitro* microglia target genes. **(E)** We also included an unbiased analysis where all ligands of the NicheNet database were used.

**Figure S3. Ligand-target network matrix of the top-ranked ligands that potentially regulate genes downregulated in *in vitro* microglia compared to *ex vivo* microglia.** NicheNet’s ligand-target matrix denoting the regulatory potential between ligands expressed by **(A)** neurons, **(B)** astrocytes, **(C)** oligodendrocytes or **(D)** microglia, and significantly downregulated *in vitro* microglia target genes. **(E)** We also included an unbiased analysis where all ligands of the NicheNet database were used.

**Figure S4. Predicted ingenuity pathways analysis (IPA) interactions of HMGB2 and its target genes.**

**Figure S5. Predicted ingenuity pathways analysis (IPA) interactions of IL-1β and its target genes.**

**Figure S6. Activity of ICM and IL-1Ra in *in vitro* primary microglia. (A)** *In vitro* primary microglia were cultured in the absence or presence of 5 µM inflachromene (ICM), and at day 14 of culture exposed for 16 h to 100 ng/mL lipopolysaccharide (LPS). mRNA expression levels of IL-6, IL-12p40 and TNF-α were analyzed. n=1. **(B)** *In vitro* primary microglia were cultured in the absence or presence of 250 ng/mL IL-1 receptor antagonist (IL-1Ra) and at day 14 of culture exposed for 16 h to 10 ng/mL interleukin (IL)-1β. mRNA expression levels of IL-6 were analyzed. Each symbol represents a donor, paired t-test on log-transformed data, n=3, * < p 0.05. Relative gene expression was standardized to housekeeping gene ACTB.

**Figure S7. Gene expression values in counts per million (CPM) of microglia signature genes in monocultured microglia and in spheres.**

**Figure S8. Predicted ingenuity pathways analysis (IPA) interaction of TGF-β3 and FOS.**

**Figure S9. Gene expression values in counts per million (CPM) of matrisome genes in *ex vivo* microglia and *in vitro* microglia.**

**Table S1. Ligands of interest.**

| **Ligands of interest** |
| --- |
| BMP2 |
| BMP7 |
| CTGF |
| FGF2 |
| HMGB2 |
| IL1B |
| PCDHGA7 |
| SPP1 |
| TGFB1 |
| YARS |
