# Supplementary figures and images for: In silico-in vitro modeling to uncover cues involved in establishing microglia identity: TGF-β3 and laminin can drive microglia signature gene expression
Source: Front Cell Neurosci. 2023 Jun 26;17:1178504. doi: 10.3389/fncel.2023.1178504 (PMC10330817; doi:10.3389/fncel.2023.1178504)

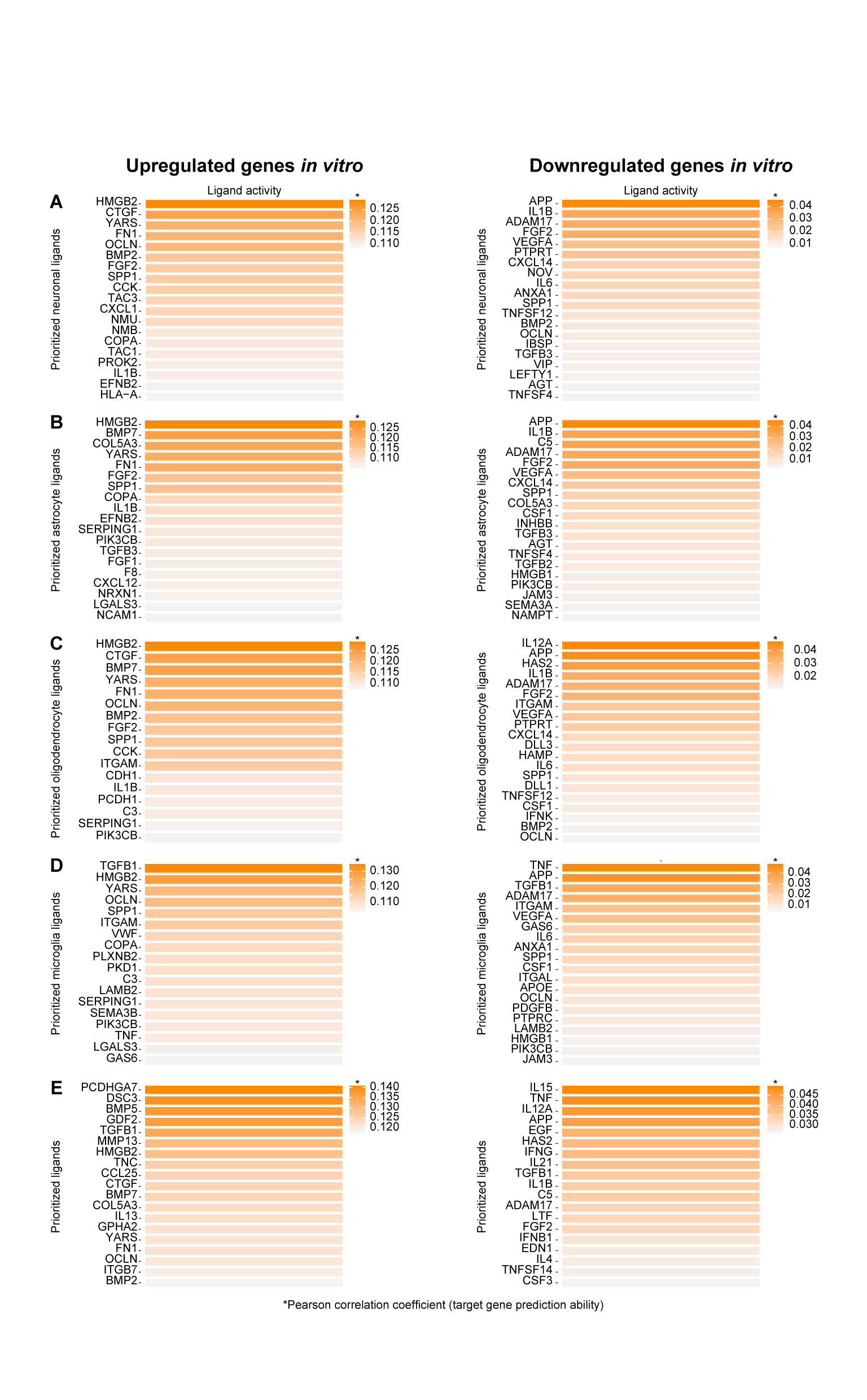

Supplement: Supplementary file 2 [file Image_1.TIF]

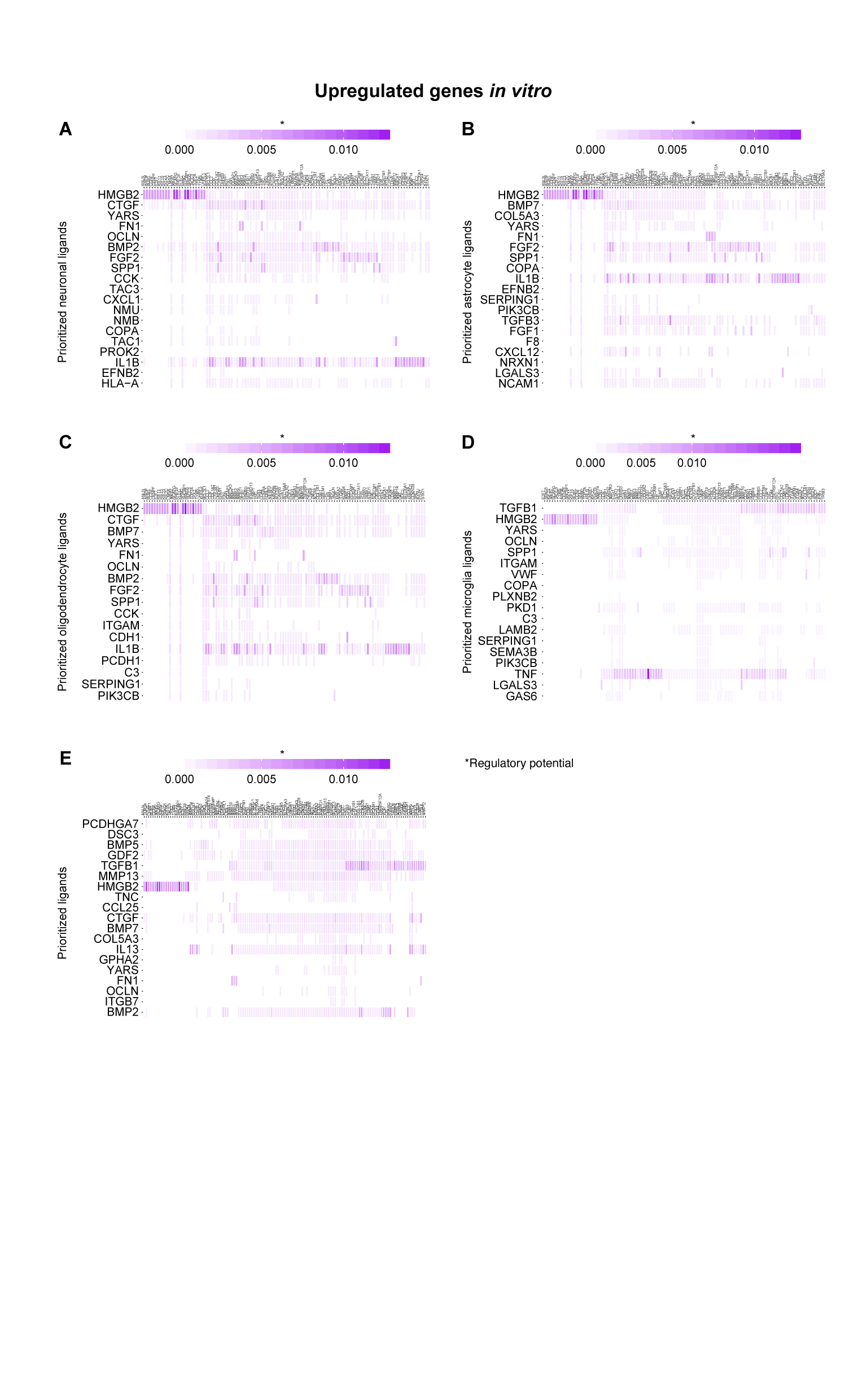

Supplement: Supplementary file 3 [file Image_2.TIF]

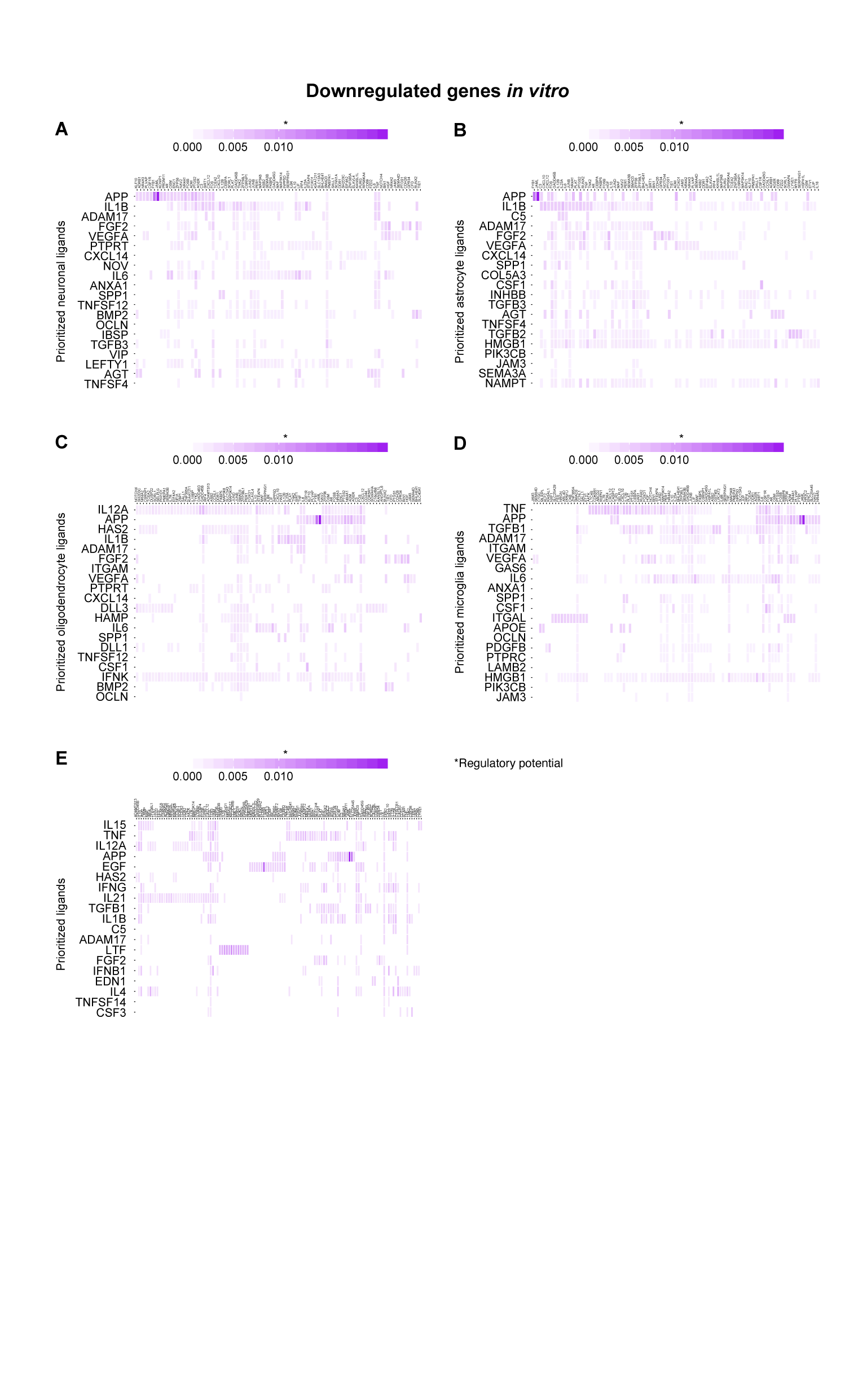

Supplement: Supplementary file 4 [file Image_3.TIF]

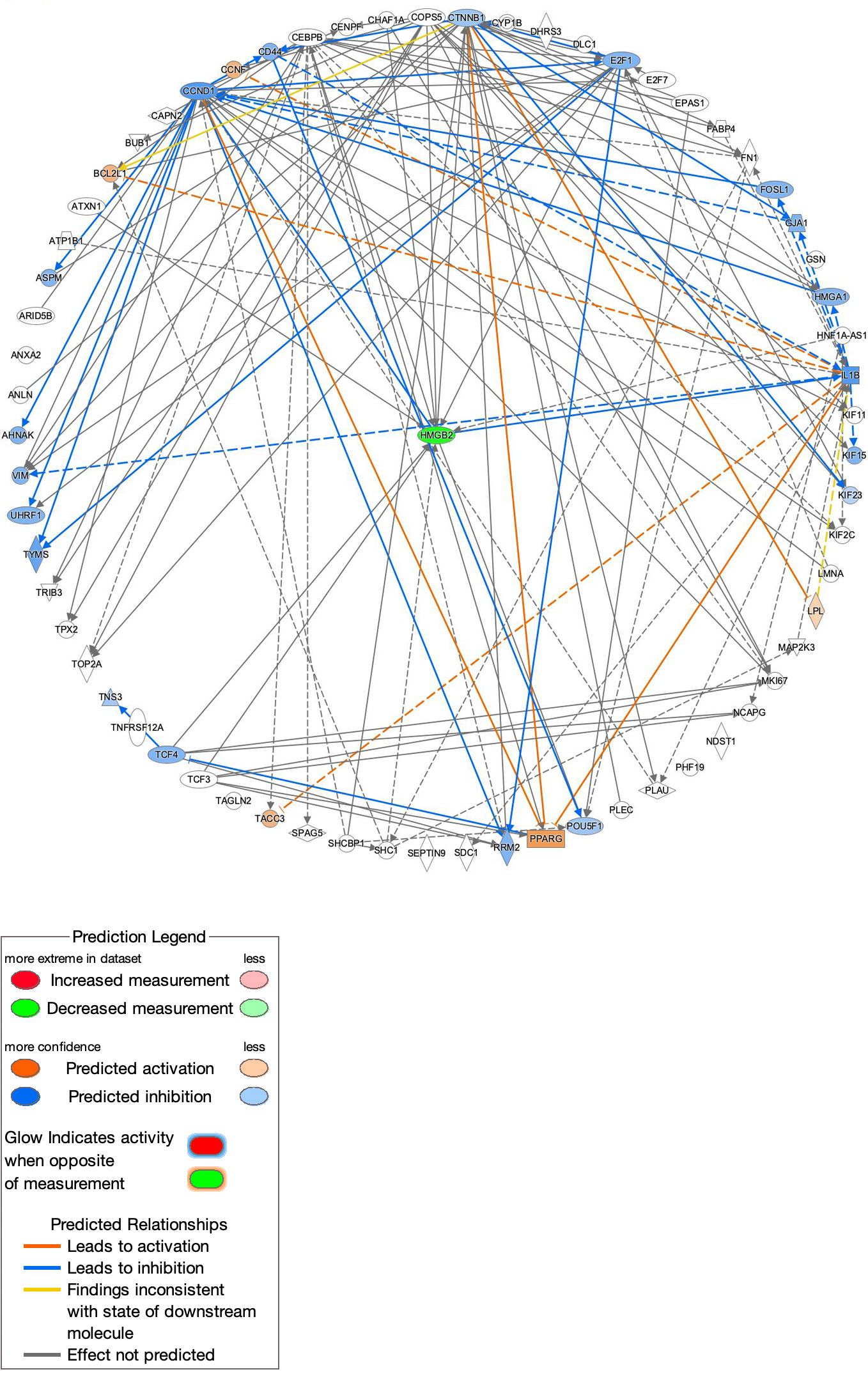

Supplement: Supplementary file 5 [file Image_4.TIF]

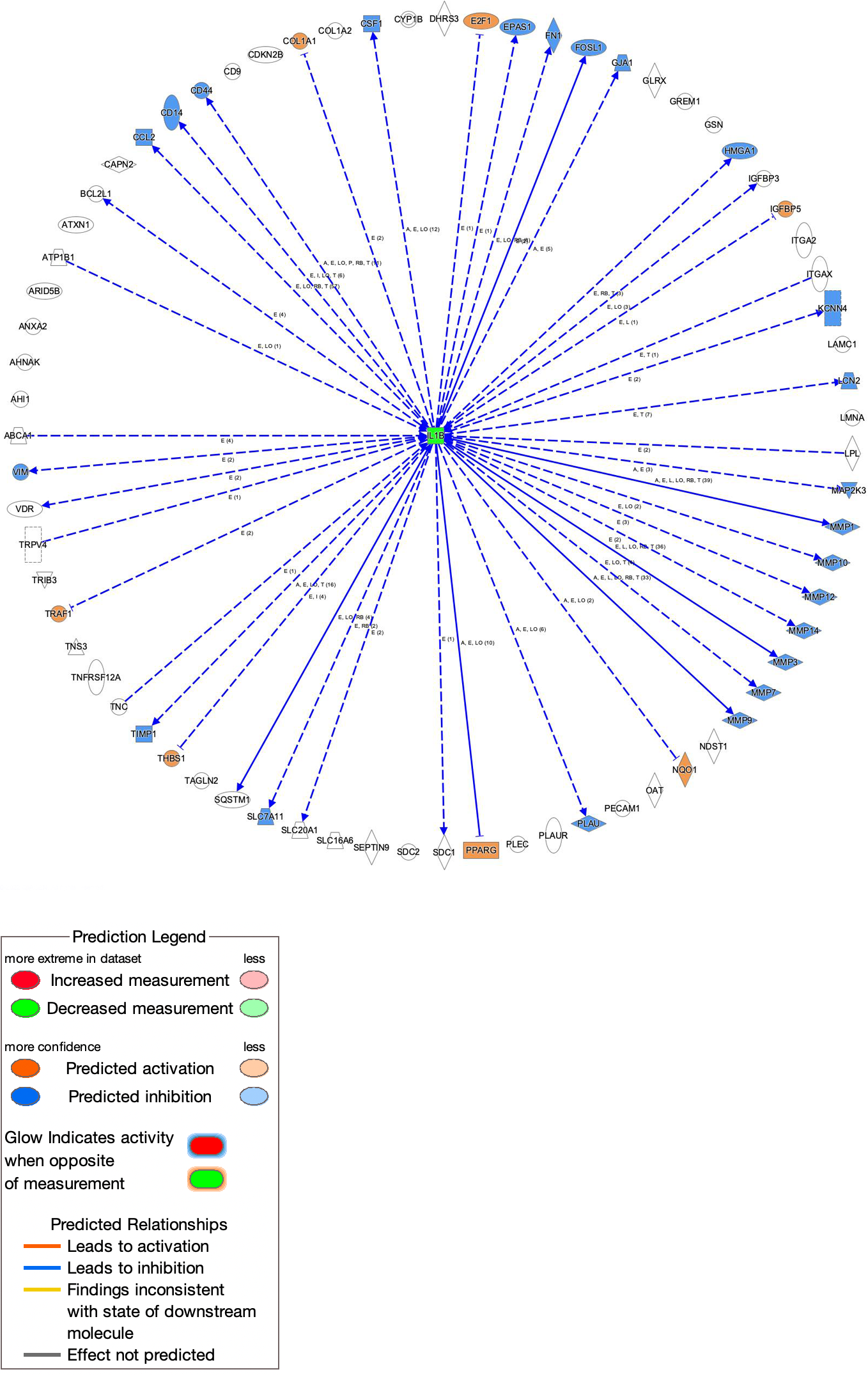

Supplement: Supplementary file 6 [file Image_5.TIF]

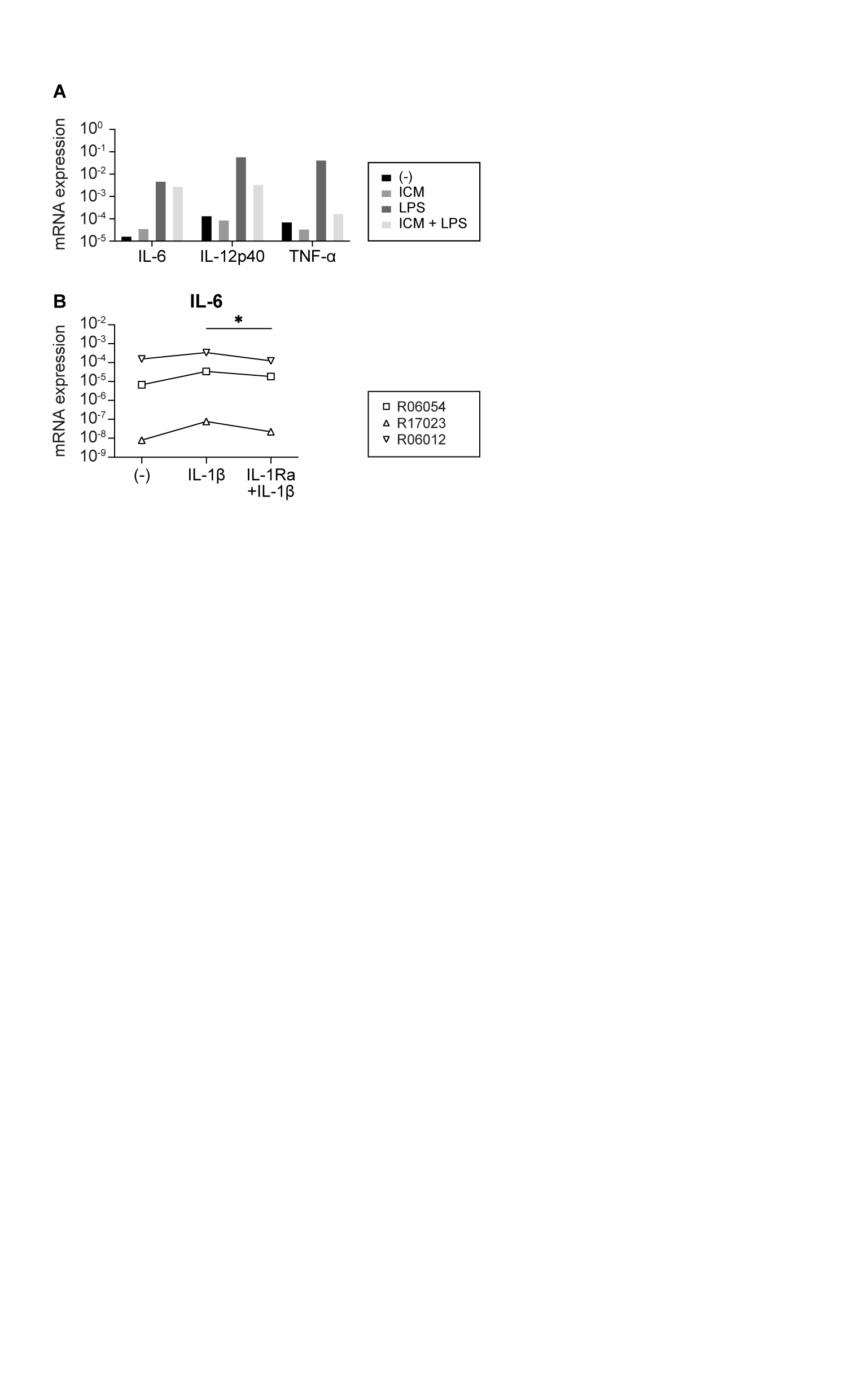

Supplement: Supplementary file 7 [file Image_6.TIF]

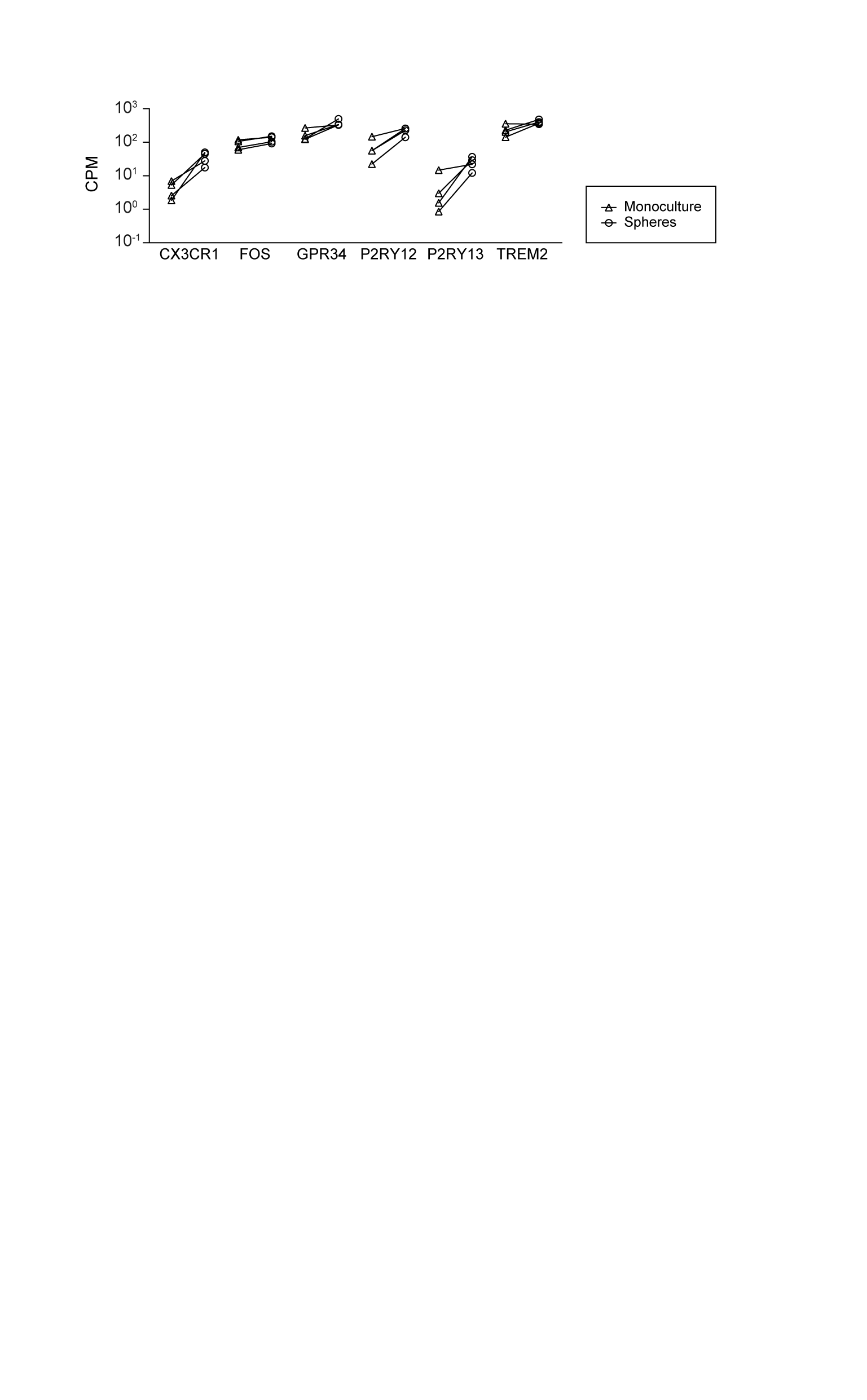

Supplement: Supplementary file 8 [file Image_7.TIF]

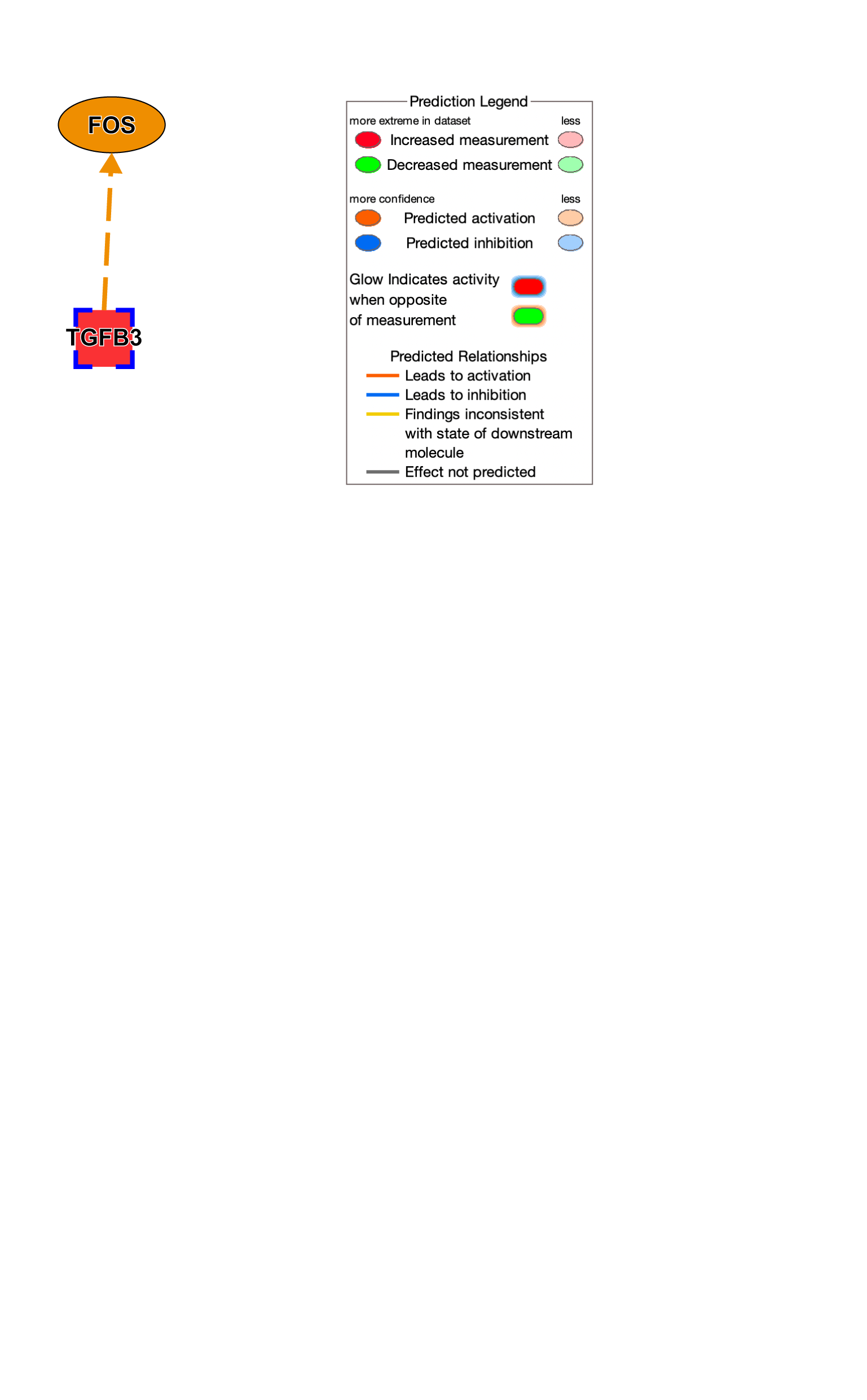

Supplement: Supplementary file 9 [file Image_8.TIF]

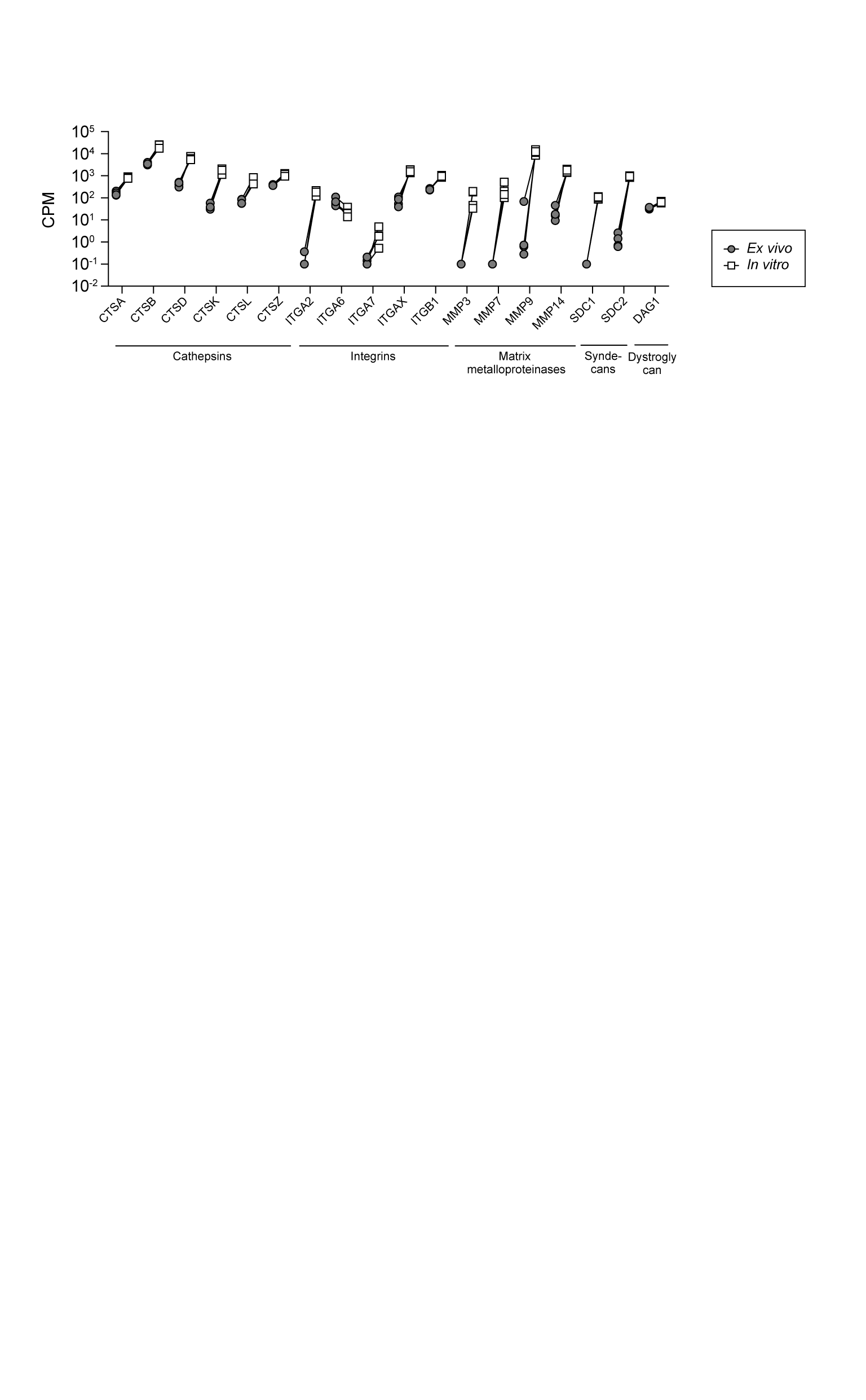

Supplement: Supplementary file 10 [file Image_9.TIF]
